# Temozolomide induces apoptosis and senescence in glioma cells cultured as multicellular spheroids

**DOI:** 10.1038/sj.bjc.6600711

**Published:** 2003-02-10

**Authors:** W Günther, E Pawlak, R Damasceno, H Arnold, A J Terzis

**Affiliations:** 1 Department of Neurosurgery, Medical University of Lübeck, Ratzeburger Allee 160, 23538 Lübeck, Germany

**Keywords:** glioma, methylation, spheroid model, senescence, apoptosis, invasion

## Abstract

Temozolomide is an alkylating cytostatic drug that finds increasing application in the treatment of melanoma, anaplastic astrocytoma and glioblastoma multiforme. The compound is a prodrug that decomposes spontaneously, independent of an enzymatic activation step. DNA methylation induces futile mismatch repair cycles and depletion of the DNA repair enzyme *O*^6^-methylguanine-DNA methyltransferase should then initiate programmed cell death. We show drug-dependent inhibition of tumour growth in a three-dimensional cell culture model of the glioma cell lines U87MG and GaMG. Migrational behaviour of the glioblastoma cells remained unaltered. However, coincubation of tumour spheroids with primary brain aggregates showed reduced tumour cell invasion into brain tissue in the presence of temozolomide. This was not achieved by slowing cellular migration, as temozolomide-treated cells displayed no reduced motility. By transferase-mediated dUTP nick-end labelling (TUNEL) of apoptotic nuclei, we found that the drug was able to induce apoptosis throughout the tumour cell spheroids. Apoptosis was highest in the core region of the spheroids. Repetitive application of sublethal doses of temozolomide to multicellular spheroids resulted in the development of drug resistance in GaMG cells. We suggest that temozolomide is a strong initiator of apoptosis in glioblastoma tumour cells in a spheroid cell culture system, when cells are already in a stressful environment.

Patients with high-grade glioma have a poor prognosis. Surgical removal of the tumour constitutes a first-line therapy. Unfortunately, glioblastoma tumour cells are highly mobile and infiltrate the surrounding, otherwise healthy, brain tissue. For this reason, surgery has to be followed by radiation and in most of the cases by chemotherapy to further lower the number of remaining tumour cells. Most chemotherapeutic agents tested to date show only marginal, if any, clinical benefit. Temozolomide is a recent addition to the chemotherapeutic arsenal, and phase III clinical trials for temozolomide treatment of recurrent high-grade glioblastomas have shown responses to the drug, reflected in an increased progression-free survival (Bower *et al*, 1997; Paulsen *et al*, 1999; Janinis *et al*, 2000; Yung *et al*, 2000)

The alkylating agent temozolomide is a second-generation imidazotetrazine prodrug. It can be administered orally and has a bioavailability of almost 100%. The compound spontaneously hydrolyses at physiological pH into the active degradation product 5-(3-methyltriazen-1-yl)imidazole-4-carboxamide (MTIC) (Tsang *et al*, 1991; Denny *et al*, 1994). MTIC methylates DNA at nucleophilic centres like N^7^-G≫N^3^-A>N^1^-A=N^3^-G=*O*^6^-G to give an order of reactivity (Friedberg, 1985). The *O*^6^-G-alkylation is reversed by the *O*^6^-methylguanine-DNA methyltransferase (MGMT) in a reaction that leads to irreversible inactivation of the enzyme protein. As MGMT is consumed by the demethylation reaction, temozolomide is thought to deplete its own detoxification mechanism. Furthermore, cotreatment of tumours with *O*^6^-benzylguanine, another otherwise nontoxic inhibitor of MGMT, increases the sensitivity of MGMT positive cells towards temozolomide (Dolan *et al*, 1990; Tentori *et al*, 1997).

In cells deficient in MGMT, *O*^6^-methylguanine is not repaired and results in the incorporation of a thymine (T) residue in the complementary position at the next replication cycle. Tumour cells that possess a functional postreplicative DNA mismatch-repair (MMR) system remove the T residue, but then reinsert again a T residue. In treated lymphoblastoid cells, several futile repair cycles are then thought to accumulate p53 protein (D'Atri *et al*, 1998; Tentori *et al*, 1998). An identical mechanism is also assumed for glioma cells (Hirose *et al*, 2001). As a consequence of this, efficient treatment of tumour cells requires, first, a functional postreplicative MMR system and, second, a functional p53 protein. Cells deficient in one of these components should show decreased sensitivity against alkylating drugs and will continue to divide until the occurrence of mitotic catastrophe. We have chosen two glioma cell lines to study the effects of temozolomide on glioma spheroid cultures. The U87MG cell line contains a functional p53 protein and was described to be sensitive to cytostatic drugs (Li *et al*, 1997), while the GaMG cell line was described to be relatively resistant to these compounds (Terzis *et al*, 1997).

Most of the studies on temozolomide and its activity against glioma cells were carried out in monolayer cultures (Sankar *et al*, 1999; Hirose *et al*, 2001). The spheroid model is a three-dimensional cell culture system that more closely resembles the *in vivo* situation inside a tumour. Along the axis of spheroids, steep gradients can exist for cellular oxygen levels, glucose concentration, nutrients, serum-derived growth factors and pH (Mueller-Klieser, 1987). Individual tumour cells growing under these conditions face a different environmental situation depending on their position inside the three-dimensional framework of the spheroid.

Moreover, the coculture model of foetal brain-derived brain aggregates and glioma spheroids offers a permissive substrate to investigate glioma cell invasion. We applied this model to investigate the effects of temozolomide on invasion.

It was our aim to study the effects of temozolomide on tumour cell migration, proliferation and invasion in a well-defined three-dimensional spheroid culture system. In addition, we asked whether glioblastoma cells grown as spheroids are able to undergo temozolomide-induced apoptosis or employ mechanisms of survival and resistance.

## MATERIALS AND METHODS

### Chemicals and drugs

Temozolomide was obtained from Essex Pharma (Munich, Germany). The drug was added to the culture medium and diluted serially prior to application. The human U87 cell line was obtained from Dr J Pontén, University of Uppsala, Sweden. The GaMG cell line was kindly supplied by Dr R Bjerkvig, University of Bergen, Norway (Akslen *et al*, 1988). Both cell lines were grown in Dulbecco's modified Eagle's medium (DMEM) supplemented with 10% heat-inactivated newborn calf serum, 2% L-glutamine, 3.2% nonessential amino acids (alanine, asparagine, aspartic acid, glutamic acid, glycine, proline, serine), penicillin (100 IU ml^−1^) and streptomycin (100 *μ*g ml^−1^) and kept in standard tissue culture conditions.

### Proliferation assay

Multicellular spheroids were aggregated from single-cell suspension and grown for 7–10 days in liquid overlay culture. Equally sized spheroids with diameters of about 250 *μ*m were placed in 24-well culture dishes (NUNC, Roskilde, Denmark) base-coated with 0.8% agar. Spheroidal volume growth was monitored daily and spheroid volume was calculated using the equation


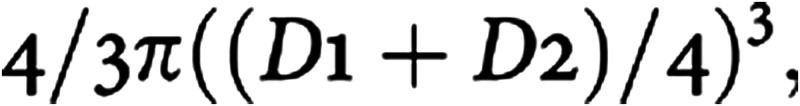
, where *D*1 and *D*2 are the maximal diameters of the spheroids measured in rectangular directions.

Temozolomide was applied in a single dose in concentrations ranging from 0.1 to 20 *μ*g ml^−1^ (0.5 *μ*M–100 *μ*M) to eight individual spheroids per temozolomide concentration. The drug was not removed after application as the half-life of temozolomide and the first derivative MTIC is about 2 h (Kim *et al*, 1997).

Drug resistance because of dealkylating MGMT enzymatic activity was probed by coincubating spheroids in 0.1 and 5 mg l^−1^ temozolomide in combination with 1 and 5 mM *O*^6^-benzylguanine, or with *O*^6^-benzylguanine alone.

To study the spheroidal response to a repetitive treatment with temozolomide, spheroids were treated with 10 or 20 *μ*g ml^−1^ temozolomide and incubated for 14 days. Thereafter, equally sized spheroids were removed and again exposed to various drug concentrations ranging from 0.1 to 20 *μ*g ml^−1^. Three independent experiments were carried out.

### Cellular migration

After 7 days of stationary culture in agar base-coated dishes, individual multicellular spheroids were placed in 24-well culture dishes. Upon adherence to the solid support, spheroids disassembled and released cells migrated away radially from their initial position. The area covered by cells was measured every 24 h over a period of 4 days. The area covered by the cells was taken as an indicator of cellular migration ability. Eight glioma spheroids were exposed to each temozolomide concentration in three independent experiments.

### Spheroid confrontation studies

Foetal brain aggregates of rat foetuses at embryonal day 18 were prepared as described by Bjerkvig *et al*. (1986). Briefly, brains were removed, minced with scalpel blades and serially treated with diluted trypsin solution. Brain aggregates formed in agar base-coated 24-well dishes and were matured over 3 weeks. Equally sized brain aggregates and glioblastoma cell spheroids were juxtaposed in agar base-coated 96-well culture dishes. Temozolomide was added after the two spheroids adhered. Confrontation cocultures were documented daily on an inverse photomicroscope. Four independent experiments with six confrontations per temozolomide concentration were carried out.

### TUNEL assay

Dewaxed sections of temozolomide-treated spheroids were boiled briefly in 10 mM citrate buffer, pH 6.0. After blocking nonspecific labelling with PBS containing 2% BSA, 0.5% NP40, sections were incubated in a terminal deoxynucleotidyl transferase-mediated dUTP nick-end labelling (TUNEL) reaction solution containing 9 mM dUTP, 1 mM DIG-labelled dUTP (Roche, Mannheim Germany), 2.5 mM cobalt chloride, 100 mM Tris pH 7.6 and 0.3 U *μ*l^−1^ terminal deoxynucleotidyl transferase. After 1 h at 37°C in a humidified atmosphere, the sections were washed twice in PBS, incubated with an anti-DIG-horseradish peroxidase conjugate in a 1 : 200 dilution (Roche, Mannheim, Germany). Unbound anti-DIG-horseradish peroxidase conjugate was removed by extensive washes. TUNEL positive nuclei were visualised by nickel-enhanced diaminobenzidine staining. Three independent experiments were carried out. Five slides of each 20 *μ*g ml^−1^ and untreated control sections were stained. Positively staining nuclei of 50 spheroidal sections per condition were counted and related to the total number of nuclei.

### DNA fragmentation

Multicellular spheroids were harvested 2 and 7 days after drug treatment. Spheroids were homogenised by five freeze–thaw cycles. Nuclei were collected by centrifugation at 1000 **g** for 10 min. Supernatant was removed and protein content was quantified by bicinchoinic-acid assay (Pierce). Nuclei were incubated in 70% ethanol at −20°C overnight. Low molecular weight DNA was extracted according to the method described by Gong (Gong *et al*, 1994). Briefly, nuclei were centrifuged at 800 **g** for 5 min, the ethanol was removed and nuclei were resuspended in 200 *μ*l 0.1 M sodium phosphate, 0.1 M sodium citrate buffer, pH 7.4 0.02% NP40, and 75 *μ*g ml^−1^ RNAse A was added and incubated for 1 h. 150 U proteinase K was added and incubated for 2 h. Fractions of the solution normalised to the protein content of the supernatant were loaded on a 1.5% TAE-agarose gel, run at 0.5 V cm^−1^ for 5 h, stained by ethidium bromide and documented.

### Senescence detection

Senescent cells were identified according to the method described by Dimri *et al*. (1995). Briefly, temozolomide-treated spheroids were washed twice in PBS, fixed in 2% paraformaldehyde, 0.2% glutardialdehyde in PBS for 5 min, washed again in PBS and incubated overnight at 37°C in a staining solution containing 1 mg ml^−1^ X-Gal, 20 mM potassium ferricyanide, 20 mM potassium ferrocyanide, and 2 mM MgCl_2_ in 40 mM citric acid/sodium phosphate buffer at pH 6.0. Subsequently, spheroids were washed, dehydrated and paraffin embedded, sectioned and counterstained with nuclear fast red and documented using a Leica Aristoplan microscope.

## RESULTS

### Proliferation and migrational characteristics

**Figure 1:**
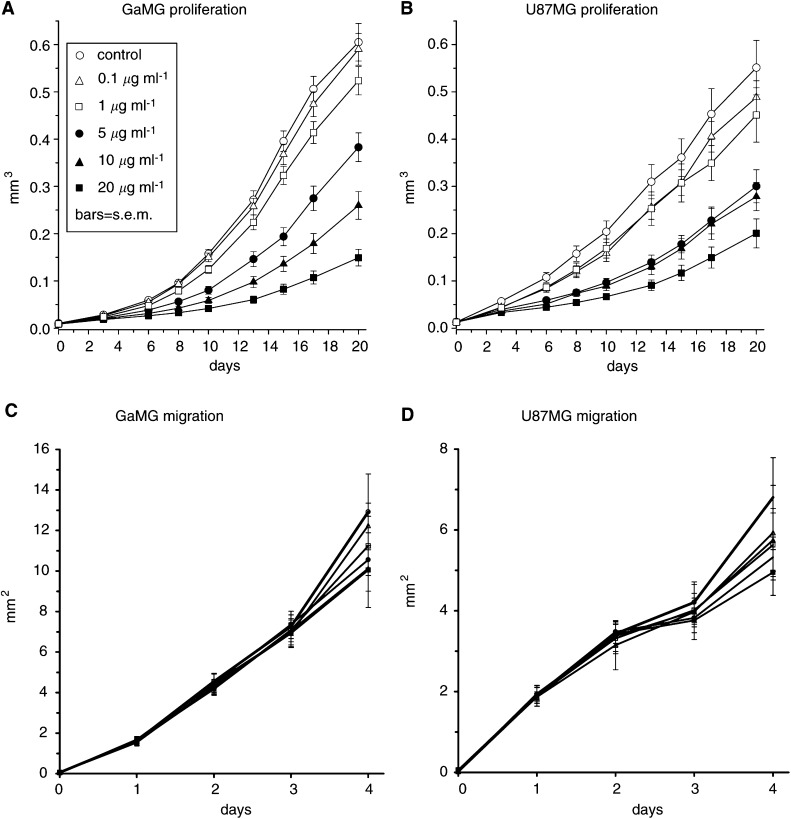
Volume growth of (**A**) GaMG and (**B**) U87MG spheroids. Spheroids treated with temozolomide show a concentration-dependent inhibition of volume growth. Error bar=s.e.m. (**C**, **D**) Radial migration of tumour cells. Area covered by cells migrating away radially from disassembling (**C**) GaMG spheroids and (**D**) U87 spheroids after placement on an uncoated cell culture dish. No difference in migration ability was observed between treated and control cells. After 5 days, differences of proliferative activity add to the migrational behaviour. Error bar=s.d.

Spheroids derived from the two glioblastoma cell lines U87MG and GaMG were exposed to increasing concentrations of temozolomide and showed a concentration-dependent relative growth inhibition (Figure 1A, B

). Growth kinetics of spheroids from both cell lines displayed an exponential growth phase. GaMG spheroids grew faster than U87MG and reached a plateau phase of growth after 16 days of culture. At this time point, U87 spheroids were still growing exponentially. While GaMG spheroids were more resistant to the cytotoxic effects of temozolomide, U87MG-derived spheroids displayed an increased sensitivity towards temozolomide. As expected, the migrational behaviour of both glioblastoma cell lines was unaffected by temozolomide (Figure 1C, D). As shown by the radial migration assay, tumour cells from a disassembling spheroid did not show a concentration-dependent inhibition of locomotor characteristics. Only prolonged incubation with temozolomide resulted in reduced radial migration, presumably because of general reduction of cell number related to the antiproliferative action of temozolomide.

### Tumour cell invasion

**Figure 2:**
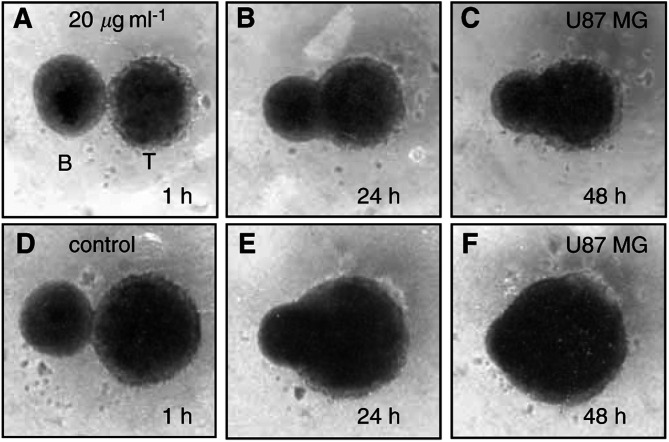
Invasion into foetal brain aggregates. Confrontation of U87MG spheroids (T) with a 21-day-old foetal rat brain aggregate (**B**). (**A–C**) Volume growth of the tumour spheroids is noticeably reduced at 20 *μ*g ml^−1^ temozolomide concentration compared to untreated controls (**D–F**). As a consequence of this, reduced invasion capacity is observed.

In cocultures between GaMG and U87MG spheroids, and mature foetal rat brain aggregates, a marked replacement of brain tissue by invading glioma cells was observed (Figure 2

). In the confrontation zone, the outer fibrous layer of glial cells was lost and the brain aggregate volume was noticeably reduced. The tumour spheroids were treated with temozolomide 3 days before confronting them to brain aggregates. As shown in Figure 1A and B, this period was long enough to induce growth inhibition. Treated tumour spheroids showed a reduction in the invasion velocity, but no change of invasion pattern was observed (Figure 2A–F).

### Acquired resistance after repetitive temozolomide administration

To mimic the repetitive application of temozolomide in clinical treatment, we applied temozolomide twice in 14-day intervals. It was our intention to get an estimate of the tumour cells' ability to develop resistance mechanisms against temozolomide. In the first round of treatment, U87 and GaMG spheroids were exposed to either 10 or 20 *μ*g ml^−1^ temozolomide. In the second round of treatment, 14 days after the first application, temozolomide in concentrations ranging from 0.1 to 20 *μ*g ml^−1^ was applied and volume growth was compared to untreated, equally sized control spheroids. While U87 MG spheroids did not survive a second application, GaMG spheroids displayed an increased resistance to temozolomide irrespective of whether the initial treatment was at 10 or 20 *μ*g ml^−1^ temozolomide. The IC_50_ values for GaMg at day 8 after the second treatment with temozolomide were above 20 *μ*g ml^−1^ (Table 1

**Table 1:** Repetitive treatment of multicellular glioblastoma spheroids with temozolomide

	**Second treatment**		
**First treatment**	**IC_50_ after 8 days**	**IC_50_ after 13 days**	**IC_30_ after 13 days**
Untreated	9.8±1.3 *μ*g ml^−1^	14.0±1.6 *μ*g ml^−1^	5.7±1.9 *μ*g ml^−1^
10 *μ*g ml^−1^ temozolomide	Not reached	Not reached	13 *μ*g ml^−1^
20 *μ*g ml^−1^ temozolomide	Not reached	Not reached	13 *μ*g ml^−1^

IC_50_ and IC_30_ values were calculated by the relation of spheroid volume growth between treated and untreated spheroids after the second treatment with temozolomide

). Control spheroids at this time point gave an IC_50_ value of 9.8±1.3 *μ*g ml^−1^. Upon extended incubation, 13 days after the second treatment, an IC_50_ could not be reached anymore. An IC_30_ of 5.7±1.9 *μ*g ml^−1^ for control GaMg spheroids, 13 *μ*g ml^−1^ for 10 *μ*g ml^−1^ pretreated GaMG spheroids and 13 *μ*g ml^−1^ for 20 *μ*g ml^−1^ pretreated GaMG spheroids was determined. In general, repetitive drug application leads to spheroid volume growth reduction and to a fast development of drug resistance.

### TUNEL in sections of spheroids

**Figure 3:**
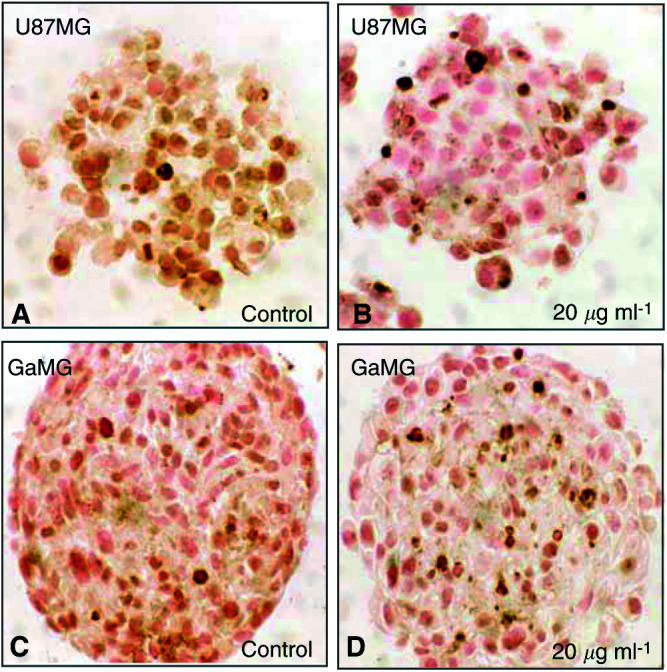
TUNEL labelling. Cross-sections of paraffin-embedded spheroids, stained for fragmented DNA by TUNEL. The more loosely associated U87 spheroids contain significantly more apoptotic nuclei 2 days after temozolomide treatment (**A**, **B**). It takes about 6 days until there is a significant number of apoptotic nuclei labelled in GaMG spheroids (**C**, **D**). Nuclei are counterstained with nuclear fast red.

Apoptosis as a result of cytostatic drug application was seen in both cell lines employed. Labelling of degraded DNA in apoptotic nuclei gives an estimate of the cytotoxic action of the drug. Distribution of apoptotic nuclei in multicellular spheroids of both cell lines showed a tendency towards an accumulation of apoptotic cells in the central region of the spheroids. To distinguish between apoptosis and necrosis, untreated control spheroids with similar diameters as the treated spheroids were also stained by the TUNEL assay. These control spheroids neither showed a central necrosis nor a significant number of TUNEL positive nuclei (Figure 3A, C

). The GaMG cell line showed only a slight induction of apoptosis as indicated in Figure 3D, while the U87 cell line was strongly driven into the programmed cell death with a high percentage of apoptotic nuclei per section (Figure 3B). After 2 days, the percentage of TUNEL positive cells was 10.7±2.4% for the U87 spheroids treated with 20 *μ*g ml^−1^ temozolomide, but almost no apoptotic nuclei were found in untreated U87 spheroids (Figure 3C). The 20 *μ*g ml^−1^ temozolomide-treated GaMG spheroids contained 2.3±0.3% TUNEL positive nuclei, while the respective control spheroids were almost without TUNEL positive nuclei; *P*<0.01, *t*-test, relative number of TUNEL positive nuclei compared to untreated control.

### DNA fragmentation after temozolomide treatment

**Figure 4:**
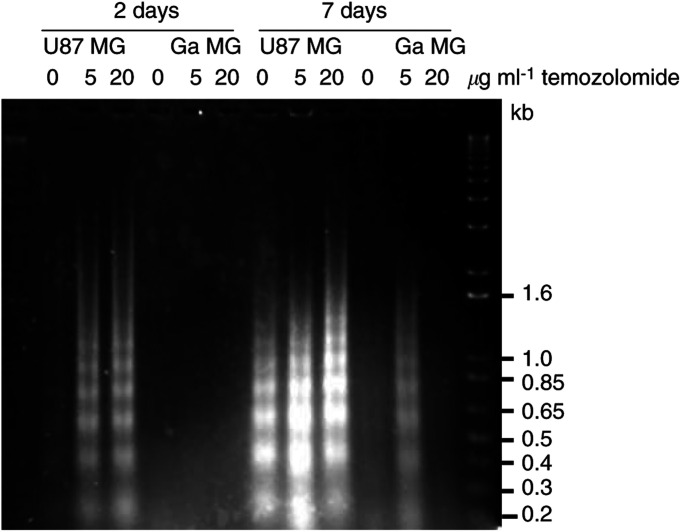
DNA fragmentation. Laddering phenomenon of fragmented DNA resolved on a 1.5% agarose gel. Alkylation of DNA by temozolomide results in induction of apoptosis in U87 cells during the first 48 h postexposure. After 7 days, untreated U87 cells also undergo apoptosis because of unfavourable culture conditions. GaMG is not initiating apoptosis after 2 days and a slight induction is observed after 7 days in culture.

To confirm that positive staining of cell nuclei in treated spheroids was because of apoptosis and not to necrosis, we performed a DNA fragmentation analysis. We observed a strong induction of the typical DNA degradation pattern as a result of apoptosis in U87 spheroids (Figure 4

). At 2 days after treatment, a drug-dependent induction of apoptosis was observed. GaMG spheroids at this time point showed no laddering phenomenon. At a later stage, 7 days after treatment, apoptosis in U87 spheroids was also prominent in untreated control spheroids. A further increase in DNA fragmentation could still be observed in temozolomide-treated U87 spheroids. GaMG spheroids did not show a significant DNA laddering phenomenon. Only spheroids treated with 5 *μ*g ml^−1^ exhibited a slight signal 7 days after treatment. These findings correspond well with the results obtained in the TUNEL assay.

### Senescence

**Figure 5:**
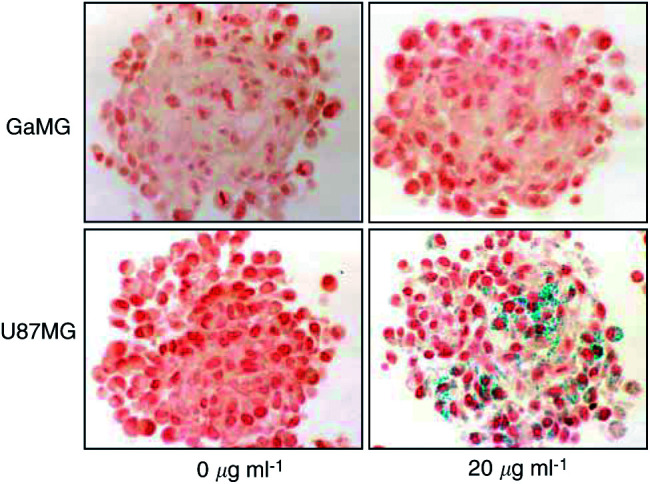
Senescence-associated galactosidase activity in glioma spheroids treated with temozolomide. X-Gal staining revealed that GaMG spheroids never accumulate a detectable galactosidase activity as a response to temozolomide treatment. However, U87 cells clearly exhibit a drug-dependent stimulation of galactosidase activity. This accumulation of lysosomal galactosidase enzyme is already observed shortly after drug application.

Cellular senescence is characterised by accumulation of lysosomal enzymes. An intense staining because of lysosomal galactosidase activity in spheroids derived from U87 cells was found. The punctuate label, because of staining of lysosomal vesicles (Kurz *et al*, 2000), was observed only after the application of temozolomide. In contrast to this, the GaMG cell line did not show any galactosidase positive intracellular vesicles. In this case, temozolomide did not induce an accumulation of galactosidase positive vesicles (Figure 5

) and the senescent phenotype. This absence of the senescent phenotype in GaMG cells may also explain the steeper slope of growth curves observed for GaMG spheroids compared to U87 spheroids.

## DISCUSSION

In the present work, the activity of temozolomide on glioblastoma cells grown in multicellular spheroids was investigated. To some extent, the three-dimensional spheroidal cell culture mimics the *in vivo* situation more closely than two-dimensional culture systems and is suitable for *in vitro* testing of cytotoxic substances (Terzis *et al*, 1997,^1998^). Cells inside a spheroid are facing respiratory stress, lack of nutrients and lack of growth factors. Reports describing an attenuated response of glioblastoma cells to temozolomide treatment (Sankar *et al*, 1999; Hirose *et al*, 2001) and a resistance of those cells to undergo apoptosis (Hirose *et al*, 2001) prompted us to investigate the drug's ability to induce programmed cell death in this cell culture model. In p53 proficient lymphoblastoid tumour cells, it has been shown that temozolomide application (D'Atri *et al*, 1998) leads to accumulation of p53 and, further downstream along the apoptotic pathway, to the specific DNA degradation pattern.

Owing to penetration difficulties of reporter dyes, the *in vivo* testing of viability by conventional assays (MTT-assay, Live–dead-assay (Molecular Probes)) could not be achieved. Therefore, we had to rely on TUNEL staining of tissue sections to determine the number of apoptotic cells in the spheroids. We found that glioblastoma cell line U87MG, which harbours a functional p53 gene (Van Meir *et al*, 1994), showed a classical apoptosis pattern already 2 days after the application of the alkylating drug. In our study, the sensitivity of the U87 cell line towards temozolomide was higher and the growth rate significantly lower than that of the GaMG cell line. Untreated control spheroids of GaMG grew significantly faster than U87 spheroids, an observation that may be because of a higher sensitivity to environmental stress factors in U87 cells. An alternative strategy to circumvent apoptosis is the prolongation of the G_2_-M arrest and the induction of a senescent state in tumour cells. This depends on a functional p53 protein, as has been described in U87 cells (Hirose *et al*, 2001). In contrast to their work carried out on cells grown in monolayer, we could show that this cell line is able to employ both strategies simultaneously. In U87MG cells, an intensive senescence-associated galactosidase activity is seen after temozolomide treatment, an observation we never made in GaMG cells. Hirose *et al* (2001) attributed this accumulation of lysosomal acid *β*-D-galactosidase (Kurz *et al*, 2000) to the prolonged G_2_-M arrest as a consequence of p53 accumulation. In contrast to our data, they could not show an activation of the apoptosis machinery in U87 cells. This difference could be explained by the fact that cells in the three-dimensional framework of a multicellular spheroid are exposed to several stress factors simultaneously. Moreover, these multiple stress factors can be integrated on the protein levels by the enzyme poly(ADP-ribose) polymerase (PARP). For a review see Tong *et al* (2001). This enzyme was demonstrated to be highly activated by both oxidative stress and chemotherapy treatment. It is thought that this protein plays a multifunctional role in many cellular processes, including DNA repair, recombination, cell proliferation and death, as well as genomic stability. In collaboration with p53, PARP is a powerful activator of apoptosis. Active PARP can poly-ADP-ribosylate the histone H1 and thereby facilitate DNA fragmentation (Yoon *et al*, 1996). Inhibition of PARP on the other hand increases cellular susceptibility to alkylating agents by inhibiting DNA repair at the G1 checkpoint (Tentori *et al*, 1998,^2001^).

A significant increase of temozolomide toxicity by coapplication of the MGMT inhibitor *O*^6^-benzylguanine was not seen in the two cell lines studied. This may be because of the intrinsic MGMT inactivation by temozolomide alone, which is not the case with other alkylating agents such as nitrosoureas (Kokkinakis *et al*, 2001). Alternatively, this can also be explained by low levels of MGMT protein in glioblastoma cells as a consequence of promoter silencing observed in glioblastoma cells (Esteller *et al*, 2000).

In clinical application, temozolomide is given over 5 consecutive days within an interval of 4 weeks. In our experiments, double treatment of glioblastoma spheroids with temozolomide with an intermittent recovery phase of 2 weeks resulted in the complete destruction of U87 spheroids after the second application of the drug, while GaMG cultures continued to grow and attained a fairly high resistance against the drug, albeit under a strong reduction of growth. This corroborates a recently published clinical study that showed that ‘long term’ low-dose temozolomide treatment is ineffective in glioblastoma patients (Khan *et al*, 2002).

The spheroid model furthermore allows analysing the potential of the drug to influence one of the most dangerous properties of glioblastoma cells, the invasion of neighbouring brain tissue (Terzis *et al*, 1997,^1998^). In a confrontation study, we could show that temozolomide is able to reduce the invasiveness of glioblastoma cells into foetal brain aggregates. This is due presumably to the reduction of cell number and not by reducing the motility of the glioma cells, as this cellular property remained unaffected by the alkylating substance. Although the putative harmful effect of temozolomide on brain aggregates was not assessed in detail in our study, we did not observe microscopical alterations like, for example, necrosis or disintegration. In the light of the slow volume growth during the 3-week maturation process, we can assume that only few cell divisions occur in the brain aggregates and most cells are arrested in G_0_. This stage of the cell cycle would render the cells relatively resistant to the cytotoxic effects of temozolomide.

Temozolomide is a potent inhibitor of glioma cell growth. Cells with a known functional p53 gene and a deficient MGMT detoxification system undergo either apoptosis or remain in a senescent state. Both pathways are able to reduce cellular proliferation and glioblastoma spheroid growth.

The glioblastoma cell line GaMG did not show a significant apoptotic induction and no senescence-associated galactosidase accumulation. Despite the evident lack of MGMT expression in GaMG cells, repetitive sublethal treatment with temozolomide selects extremely resistant cells.

Taken together, our study suggests that it might be important in the treatment of glioblastoma to apply temozolomide at the maximal dosage tolerated in the first cycle in order to avoid selection of resistant cells.
